# Antibiotic Resistance Identification Among the Patients with *Helicobacter pylori* Infection: A Cross-sectional Study from Iran

**DOI:** 10.34172/mejdd.2025.427

**Published:** 2025-07-30

**Authors:** Fatemeh Hasanvand, Javid Sisakhtpour, Mohamadreza Salehi, Khalil Pestehei, Sepideh Khodaparast

**Affiliations:** ^1^Department of Bacteriology, Faculty of Medical Sciences, Tarbiat Modares University, Tehran, Iran; ^2^Department of Infectious Diseases and Tropical Medicine, Tehran University of Medical Sciences, Tehran, Iran; ^3^Department of Anesthesiology and Pain, Imam Khomeini Hospital Complex, Tehran University of Medical Sciences, Tehran, Iran; ^4^Brain and Spinal Cord Injury Research Centre, Neurosciences Institute, Tehran University of Medical Sciences, Tehran, Iran

**Keywords:** *Helicobacter pylori*, Iran, Metronidazole, Clarithromycin, Amoxicillin, Tetracycline, Antibiotic resistance

## Abstract

**Background:**

The successful treatment of *Helicobacter pylori* infection is warranted only by using appropriate antimicrobial agents determined after performing antibiotic susceptibility tests. Determining the susceptibility profile of antibiotics currently used against *H. pylori* in each geographical region can be very practically beneficial for all drug prescribers.

**Methods:**

In this cross-sectional study, we aim to identify the frequency of four antibiotics mainly referred to in the battle against *H. pylori* strains isolated from gastric biopsy specimens of 260 patients with dyspepsia referred to the gastroenterology clinics in Lorestan between 2020 and 2022, marking the first report from Iran. After culturing 260 gastric biopsy samples, *H. pylori* strains were identified using standard diagnostic methods.

**Results:**

The prevalence of antibiotic resistance among *H. pylori* isolates was reported using the antibiotic disc diffusion method with metronidazole, clarithromycin, amoxicillin, and tetracycline discs. Moreover, the minimum inhibitory concentration (MIC) of tetracycline-resistant *H. pylori* strains, screened by the diffusion disc method, was determined using the E-test method. Out of 260 biopsy samples, 187 (72%) samples (male: 42%, female: 58%) were *H. pylori* positive. The prevalence of antibiotic resistance rates among 187 isolates of *H. pylori* was as follows: metronidazole (87.7%, n = 166), clarithromycin (63.1%, n = 118), amoxicillin (39%, n = 73), and tetracycline (34.2%, n = 64), accordingly. Antibiotic resistance among *H. pylori* isolates was higher than expected, especially for amoxicillin and tetracycline, which is a concerning issue.

**Conclusion:**

Consequently, considering the central role of the antibiotic susceptibility profile in experimental therapy, it is recommended that determining the resistance pattern of the bacterium in all parts of Iran can lead to an effective treatment regimen.

## Introduction

*Helicobacter pylori* is a microaerophilic and spiral microorganism causing superficial acute gastritis, peptic ulcer disease (PUD), and gastric adenocarcinoma.^1^ *H. pylori*’s lifelong colonization in the human stomach is found to be the main cause of inducing many gastroduodenal diseases and extra-digestive disorders, including anemia, B_12_ deficiency, and abnormal inflammatory diseases.^2^ The prevalence of *H. pylori* in developed countries ranges from 20% to 35%, while in developing countries, the rates are typically higher than 65%, conversely.^3^ The first exposure to *H. pylori* typically occurs in childhood, and carriers remain colonized if antibiotic therapy is not properly prescribed.^4^ The rationale for eliminating *H. pylori* is not only to remove the bacterium from the gastric micro niche but also to reduce the risk of severe gastroduodenal diseases, as mentioned above.^5,6^ Following the discovery of *H. pylori*, clinicians sought to determine the most effective formulation of antibiotic therapy against this elusive infection. Accordingly, many studies have been conducted to design and optimize the formulation of the therapy; however, it remains a challenging issue.^7^ Relatively persistent colonization of this bacterium in half of the world’s population is an inevitable initiative among gastroenterologists and bacteriologists to term *H. pylori* as the most common human bacterial infection ever. Following the expected phenomena, *H. pylori* assumed an eradicable microbe, at least within the first decade after its historical discovery.^8^ Many antibiotics used to provide an effective therapeutic regimen against *H. pylori* including amoxicillin, clarithromycin, tetracycline, and metronidazole, so far.^9^ Consequently, the purpose of this survey is to analyze the prevalence of antibiotic resistance among *H. pylori* clinical strains retrieved from Iranian patients with dyspepsia who were treated with metronidazole, clarithromycin, amoxicillin, and tetracycline for the first time in Lorestan province.

## Materials and Methods

###  Sample Collection

 Antral biopsies were collected for culture from 260 patients who underwent upper gastroduodenal endoscopy at Shohadaye Ashayer Hospital in Khorram Abad, Lorestan, Iran, from August 2020 to February 2022.

 Patients who reported using antibiotics within the 3 months preceding study entry were excluded from the analysis. Endoscopic findings and pathology data were used as criteria for the clinical diagnosis of conditions in these patients.^10,11^

###  Helicobacter pylori Culture

 Two antral biopsy specimens were taken during endoscopy - one for a rapid urease test (RUT) and the other inoculated into sterile thioglycolate broth. The broth was sent to the laboratory within 2 hours after homogenization. 100 μL of homogenate were inoculated onto Brucella agar supplemented with 7% defibrinated sheep blood, 7% fetal calf serum (FCS), and also Selectab Skirrow (MAST, UK) per 250 mL agar was added to prevent contamination. Cultures were incubated at 37 °C for 7-8 days in microaerophilic conditions provided by an anaerobic jar and gas pack (10% CO2, 5% O2, and 85% N2). Bacterial identification was confirmed using classic biochemical tests, including catalase, oxidase, urease, gram staining, and colony morphology. All isolates were stored at -80 °C in brain heart infusion broth supplemented with 15% glycerol and 7% FCS for future analysis. In summary, standard culture and identification techniques were used on biopsy specimens from patients to isolate and characterize any *H. pylori* bacteria.^12^

###  Antibiotic Susceptibility Testing

 Antibiotic resistance patterns of isolated *H. pylori* strains were determined for four antibiotics: metronidazole, clarithromycin, amoxicillin, and tetracycline. The method used to test resistance was the disk diffusion method. Disks containing the four antibiotics were obtained from MAST, UK. Resistance testing was performed by disk diffusion on Muller-Hinton agar supplemented with 7% defibrinated sheep blood and 7% FCS. The isolated *H. pylori* strains underwent antibiotic susceptibility testing for the four common antibiotics used to treat *H. pylori* infections. The disk diffusion method was utilized on supplemented Muller-Hinton agar to determine resistance patterns.^13^

 Suspensions from initial cultures were prepared in Brucella broth adjusted to a 0.5 McFarland standard turbidity. 100 μL of bacterial suspension was lawn-poured onto Mueller-Hinton agar supplemented with defibrinated sheep blood and FCS. Disks containing 5 μg of metronidazole, 2 μg of clarithromycin, 10 μg of amoxicillin, and 30 μg of tetracycline were placed on the agar surface. Plates were incubated at microaerophilic conditions at 37 °C for 5 days. Zones of growth inhibition around each disk were measured in mm. Interpretive criteria for resistance: Clarithromycin - zone < 22 mm, metronidazole - zone < 16 mm, amoxicillin - zone < 25 mm, tetracycline - zone ≤ 30 mm. In general, a standardized disk diffusion assay was used to test the *H. pylori* isolates for resistance to the four antibiotics based on defined inhibition zone diameter cutoffs (Table 1),^14-18^

**Table 1:** Growth inhibition zone for *H. pylori*

**Antibiotic**	**Susceptible**	**Intermediate**	**Resistant**
Metronidazole	≥ 21 mm	16-21 mm	< 16 mm
Clarithromycin	≥ 22 mm		< 22 mm
Amoxicillin	≥ 25 mm		< 25 mm
Tetracycline	˃ 30 mm		≤ 30 mm

 The minimum inhibitory concentration (MIC) of isolates showing reduced susceptibility by disk diffusion was determined using the E-test method. A suspension with a turbidity of 0.5 McFarland was prepared from the pure culture, and 100 microliters of it were transferred to Mueller-Hinton agar culture medium enriched with defibrinated sheep blood and FCS. After drying, E-test strips containing antibiotic gradients were placed on the media. Plates were incubated for 48 hours under microaerophilic conditions. Classification of resistance was based on CLSI guidelines: Clarithromycin MIC > 0.5 μg/mL – resistance, Metronidazole MIC > 8 μg/mL – resistance. Thus, the E-test method was used to precisely determine the MIC of isolates that showed reduced susceptibility by disk diffusion testing. Classification of resistance was according to standard CLSI breakpoints using this quantitative MIC method (Table 2).

**Table 2:** MIC breakpoint for *H. pylori*^14-18^

**Antibiotic**	**Susceptible ** **(µg/mL)**	**Intermediate ** **(µg/mL)**	**Resistant ** **(µg/mL)**
Metronidazole	≤ 4	4 < MIC < 8	≥ 8
Clarithromycin	≤1	1< MIC < 2	≥ 2
Amoxicillin	≤1	1< MIC < 2	≥ 2
Tetracycline	≤2	2< MIC < 4	≥4

Reference standard strain ATCC43504 was included as a quality control.

## Results

###  Characteristics of Patients

 Based on the updated data, the breakdown of patient characteristics is as follows: Total Number of *H. pylori* Strains: 187. Sex distribution: Female: 110 isolates (58.8%), male: 77 isolates (41.1%). Gastroscopic conditions: Gastritis: 110 strains (58.8%), stomach ulcer: 37 strains (19.7%), duodenal ulcer: 17 strains (9%). Cancerous samples: 10 strains (5.3%). No endoscopic symptoms: 13 strains (6.9%). It is important to note that the total number of strains (187) matches the number mentioned in the provided information. The prevalence of *H. pylori* in the tested biopsies is stated to be 71.9% (Table 3).

**Table 3:** Breakdown of patient characteristicsin Lorestan Province

	**Gastritis**	**Stomach ulcer**	**Duodenal ulcer**	**Cancerous**	**No endoscopic symptoms**
Female	73	20	6	3	8
Male	37	17	11	7	5
Total	110	37	17	10	13

###  Prevalence of Antibiotic Resistance

 According to the provided information, here are the resistance levels of different antibiotics among the *H. pylori* strains:

1. Metronidazole:
  - Resistance rate: 87.7%
  - Resistance rate by disk diffusion method: 70.5%
  - Resistance rate by E-test: 70.5%
2. Clarithromycin:
  - Resistance rate by disk diffusion method: 63.1%
  - Resistance rate by E-test: 46.5%
3. Amoxicillin:
  - Resistance rate by disk diffusion method: 39.03%
  - Resistance rate by E-test: 26.7%
4. Tetracycline:
  - Resistance rate by disk diffusion method: 34.2%
  - Resistance rate by E-test: 18.7%

It is worth noting that 21 isolates were found to be susceptible to all the antibiotics examined. Additionally, the rate of resistance to metronidazole, clarithromycin, amoxicillin, and tetracycline was higher in patients with PUD compared with patients with chronic gastritis (CG) (*P* < 0.05). These findings indicate that metronidazole had the highest resistance level among the tested antibiotics, followed by clarithromycin (*P* < 0.05). On the other hand, amoxicillin and tetracycline showed relatively lower levels of resistance. The resistance rates varied between the disk diffusion method and the E-test for each antibiotic (Table 4).

**Table 4:** Antimicrobial susceptibility of *H. pylori* isolates in Lorestan province

**Antibiotic**	**Disc diffusion**			**MIC**^1^ **(µg/mL)**		
	**S**^2^	**I**^3^	**R**^4^	**S**	**I **	**R**
Metronidazole	12	9	166	43	12	132
Clarithromycin	69	_	118	88	7	87
Amoxicillin	114	_	73	132	5	50
Tetracycline	123	_	64	141	11	35

1. Minimum inhibitory concentration, 2. Sensitive, 3. Intermediate, 4. Resistant.

###  Multidrug Resistance (Disc Diffusion)

 According to the provided information, here are the observations regarding single-drug resistance (SDR) and multidrug resistance (MDR) among the *H. pylori* isolates:

✓ Single-drug resistance:
  - SDR was observed in 45 isolates (24%).
  - Resistance to metronidazole was the only SDR phenotype.
✓ Multidrug resistance:
  - MDR was observed in 49 isolates (22%).
  - Three different MDR profiles were identified.
  - The most common MDR profiles were: MTZ + CLA + AMO + TET: 30%; MTZ + CLA + AMO: 39%; and MTZ + CLA + TET: 33%.

Additionally, out of the total 187 clinical isolates of *H. pylori*, 21 strains (11.22%) were susceptible to all antibiotics tested. These findings indicate that SDR was observed in a subset of isolates, with metronidazole being the most commonly observed single-drug resistance phenotype. MDR was also observed in a significant portion of the isolates, with three different MDR profiles identified. The most common MDR profiles involved combinations of metronidazole, clarithromycin, amoxicillin, and tetracycline. However, a notable proportion of isolates remained susceptible to all antibiotics tested (Table 5).

**Table 5:** Profile of antimicrobial susceptibility

	**MDR**	**SDR**	**Sensitives**
Gastritis	11	35	18
Stomach ulcer	15	0	0
Duodenal ulcer	13	0	0
Cancerous	10	0	0
No endoscopical symptoms	0	10	3
Male	28	21	8
Female	21	24	13
Total	49	45	21

## Discussion

 For many years, there has been a general consensus among medical professionals regarding the importance of considering *H. pylori* as a pathogenic bacterium that requires prompt antibiotic therapy for effective bacterial eradication.^19^ The increasing rate of *H. pylori* elimination failure and the emergence and spread of antibiotic resistance among persistent strains make it crucial to disclose the prevalence of *H. pylori* infections and their antimicrobial susceptibility profiles. This information is essential for achieving more efficient bacterial eradication and implementing effective control measures. The prevalence of *H. pylori* infection and antibiotic resistance can vary significantly across different geographic regions. Numerous studies have reported both concordant and varying findings in this regard. Generally, the prevalence of *H. pylori* infection tends to be higher in developing countries than in developed countries. This difference can be attributed to various factors such as differences in socioeconomic conditions, hygiene practices, and access to healthcare. In developing countries, crowded living conditions, lower socioeconomic status, and limited access to clean water and sanitation facilities can contribute to a higher risk of *H. pylori* transmission and infection.

 On the other hand, developed countries often have lower *H. pylori* infection rates due to improved living conditions, higher standards of hygiene, and better access to healthcare. However, it is important to note that *H. pylori* infection can still occur in developed countries, particularly among certain populations with higher risk factors such as immigrants from high-prevalence regions. Regarding antibiotic resistance, the prevalence of *H. pylori* strains resistant to commonly used antibiotics can also vary geographically. This is influenced by factors such as regional antibiotic prescribing practices, local patterns of antibiotic resistance, and the population’s exposure to antibiotics. Some regions may have higher rates of antibiotic resistance due to inappropriate use of antibiotics, while others may have lower rates due to more prudent antibiotic prescribing practices. For example, in European countries, the prevalence of *H. pylori* antibiotic resistance is generally lower compared with some other regions. Studies have reported resistance rates below 20% for antibiotics such as clarithromycin, metronidazole, and levofloxacin in several European countries. However, it is important to note that resistance rates can still vary within Europe, and there may be pockets of higher resistance in certain populations or geographic areas.

 In contrast, some Eastern Mediterranean countries have reported higher rates of *H. pylori* antibiotic resistance. Studies have shown resistance rates above 80% for certain antibiotics, indicating a significant challenge in eradicating *H. pylori* infections in those regions.^20^ Understanding the antibiotic resistance patterns of *H. pylori* strains isolated from patients is crucial for prescribing the most effective therapy. Evaluating antibiotic resistance patterns before prescribing antibiotics can help prevent the overuse of unnecessary antibiotics and reduce medical-related costs in every region. In recent decades, the rate of *H. pylori* antibiotic resistance has increased in Iran, primarily because of the widespread use of antibiotics. This phenomenon is concerning as it can significantly impact the success of *H. pylori* eradication therapies. The overuse or misuse of antibiotics can contribute to the development of antibiotic resistance in *H. pylori* strains, making it more challenging to treat infections caused by these bacteria. A study conducted in 2005 aimed to evaluate the incidence of *H. pylori* infection in children and adolescents in different regions of Iran. The study’s findings reported an infection rate of 70% in the southwest of Iran and 32% in the northwest of Iran. These rates indicate a significant burden of *H. pylori* infection in these regions, highlighting the need for effective management strategies. The high prevalence of *H. pylori* infection in Iran, particularly in certain regions, underscores the importance of implementing appropriate diagnostic and treatment protocols. It is crucial to consider local antibiotic resistance patterns when selecting the most effective therapy for eradicating* H. pylori*. This approach helps ensure successful treatment outcomes and reduces the risk of treatment failure due to antibiotic resistance (Table 6).^20^

**Table 6:** Pattern of antibiotic resistance of *H. pylori* in different parts of Iran

**Area**	**Year**	**Sample(n)**	**Strain**	**Method**	**MTZ**	**CLA**	**AMO**	**TET**	**Reference**
Sari	2009	210	197	Disk diffusion	65.5%	42.2%	23.9%	37.1%	^21^
Elam	2009-2010	NA	50	Disk diffusion	88%	32%	NA	12%	^22^
Tehran	2009-2010	170	150	Agar dilution	78.6%	34%	10%	9.3%	^23^
Tehran	2009-2010	NA	NA	Agar dilution	60%	17%	10%	5%	^24^
Azerbaijan	2010-2011	395	112	Disk diffusion	76.8%	14.3%	28.6%	18.7%	^8^
Shiraz	2010	266	121	E. test	44%	5%	20%	3%	^25^
Tehran	2010-2011	197	111	Agar dilution	61.3%	32.4%	NA	NA	^26^
Tehran	2010-2017	985	218	Disk diffusion	79.4%	34.4%	27.1%	38.5%	^27^
Isfahan	2011-2012	260	78	Disk diffusion	55.1%	15.3%	6.4%	NA	^28^
Kerman	2011	191	63	Disk diffusion	NA	31.7%	NA	NA	^29^
Rasht	2012-2013	NA	89	E. test	NA	5.6%	NA	NA	^30^
Yazd	2012-2013	651	144	Disk diffusion	77.8%	18.8%	7.6%	21.5%	^31^
Rasht	2012-2014	169	21	Disk diffusion	57.1%	15.3%	4.8%	23.8%	^32^
Kashan	2013	246	95	E. test	NA	NA	NA	NA	^33^
Tabriz	2013	NA	123	Disk diffusion	78.68%	17.07%	27.68%	NA	^34^
Shiraz	2014	223	84	Disk diffusion	64.3%	NA	NA	NA	^35^
Tabriz	2014	NA	104	E. test	NA	NA	NA	10.6%	^36^
Tehran	2014-2015	122	55	Agar dilution	60%	NA	NA	NA	^37^
Tehran	2015	90	32	Disk diffusion	_	_	53%	25%	^38^
				Agar dilution	62.5%	22%	_	_	
Ahvaz	2015-2016	NA	157	E. test	43.94%	NA	NA	NA	^39^
Tehran	2016	78	33	Agar dilution	81.8%	36.4%	30.3%	6.1%	^40^
Tehran	2018	NA	104	Agar dilution	82.7%	35.6%	29.8%	50%	^41^
Chaharmahal va Bakhtiari	2018	650	526	Disk diffusion	61.97%	63.68%	NA	62.92%	^42^

MTZ, metronidazole; CLA, clarithromycin; AMO, amoxicillin; TET, tetracycline; NA, not available.

 According to studies, *H. pylori* has indeed infected more than half of Iranians over the past decade. *H. pylori* is a highly prevalent bacterium worldwide, and its infection rates can vary across different populations and regions.^43^ In Iran, studies have reported a high prevalence of *H. pylori* infection in the adult population, ranging from approximately 40% to 90%. This indicates that a significant proportion of Iranian adults carry the bacterium. It is widely recognized that *H. pylori* infection is often acquired early in childhood. Transmission of the bacterium can occur through person-to-person contact, primarily within families or close communities. Factors such as poor sanitation, crowded living conditions, and shared utensils or food can contribute to the spread of *H. pylori*.^38,42,43^ The study conducted in Iran for the first time reported a prevalence of 79% for *H. pylori* resistance, indicating a high rate of antibiotic resistance among *H. pylori* strains. This finding aligns with previous studies conducted in various geographical areas of Iran, suggesting an increasing prevalence of antibiotic resistance in recent years. Addressing this issue requires continued surveillance, appropriate antibiotic prescribing practices, and efforts to promote antimicrobial stewardship.

###  Clarithromycin

 Clarithromycin is widely recognized as a key antibiotic for *H. pylori* eradication therapy due to its significant impact on treatment outcomes. It is part of the standard triple therapy regimen, which typically includes a proton pump inhibitor, clarithromycin, and amoxicillin or metronidazole.^20^ In general, the rate of clarithromycin resistance in *H. pylori* strains is typically lower compared with metronidazole resistance. However, it is important to note that the rate of primary clarithromycin resistance is indeed increasing and can vary across different geographical regions.^39^ Clarithromycin resistance rates in *H. pylori* strains can vary from 30% to 50% in different parts of the world. This variation highlights the importance of considering regional resistance patterns when developing treatment strategies for *H. pylori* infections. Monitoring resistance rates and implementing appropriate measures are crucial for enhancing treatment success and combating the emergence of further resistance. The reported rates of clarithromycin resistance in different regions are approximately 27.46% in Asia, 22.11% in Europe, 5.46% in Africa, 30.8% in North America, and 12.88% in South America. These figures highlight the regional variations in clarithromycin resistance among *H. pylori* strains and emphasize the importance of tailored treatment approaches based on local resistance patterns.^27^ Resistance to clarithromycin tends to be lower (around 10%) in developed countries and higher (ranging from 25% to 50%) in developing countries. The higher rates of resistance in developing countries highlight the need for improved access to healthcare, effective surveillance, and antimicrobial stewardship programs to combat the growing problem of antibiotic resistance in *H. pylori* infections.^44^ It is noteworthy that an increase in resistance to clarithromycin in Asia from 15.28% in 2009 to 32.46% in 2014 was reported, possibly due to increased use of macrolide drugs in Asian countries. It can be stated that resistance to clarithromycin is slowly increasing worldwide. According to the resistance of *H. pylori* to clarithromycin (63.1%) in the present study, as well as previous studies conducted in Iran (Table 4), the rate of resistance to this antibiotic has been increasing in recent years. Meanwhile, the increase in resistance to this antibiotic can be associated with a decrease in the effectiveness of the treatment regimen containing clarithromycin in the successful eradication of this organism.

###  Metronidazole

 The prevalence of metronidazole resistance among Iranian *H. pylori* strains is reported to be high and varies from 40.5% to 79%. Resistance to metronidazole, which has been widely used in the treatment of *H. pylori* infection, varies from 8% to 80% in different parts of the world. Resistance to metronidazole has been reported in Asia (46.57%), Europe (31.19%), Africa (75.02%), North America (30.5%) and South America (52.85%). Metronidazole resistance in developed countries reaches approximately 30%, whereas in developing countries is much higher (60%).^45^ From the high rate of metronidazole resistance observed in this study (87.7%) and previous studies conducted in Iran (Table 6), it can be concluded that the resistance of *Helicobacter pylori* strains to metronidazole remains high in Iran. There is a correlation between high levels of metronidazole resistance and the economic conditions of the community, which can be due to the high level of consumption of this antibiotic in the treatment of infectious diseases of women, oral and dental, as well as parasitic infections.^46,47^ Therefore, based on other studies, *H. pylori* treatment regimens containing metronidazole are not effective and should not be chosen as the first-line eradication treatment in Iran.

###  Amoxicillin and Tetracycline

 Global resistance to amoxicillin, including common antibiotics in the treatment of *H. pylori* infection, has not been common and has been reported at a low level (14.67%). Resistance to amoxicillin has been reported in Asia (23.61%), Europe (0.35%), Africa (40.87%), North America (2%), and South America (6.56%). Resistance to amoxicillin varies across different regions of the world. It has been reported in Asian countries, including Malaysia (15.2%), Taiwan (15.2%), Japan (15.2%), India (72.5%), and Bangladesh (6%), but not in Vietnam (0%).^43,48^

 The overall resistance to tetracycline, which is among antibiotics commonly used in second-line *H. pylori* eradication regimens, is 11.7% worldwide.^49^ Fortunately, among the four common antibiotics used to eradicate *H. pylori* infections, tetracycline resistance is at the lowest level. Resistance to tetracycline has been determined in Asia (7.38%), Europe (7.15%), Africa (50%), and in North America and South America, it is nearly zero. Resistance to tetracycline has been reported in China (0.6%), South Korea (0.01%), India (58.3%) and Iran (12.2%).^43,44^ Studies have revealed that *H. pylori* resistance to tetracycline has increased from 6.11% in 2009 to 26.85% in 2014.^43,50,51^

 According to the level of resistance of *H. pylori* to amoxicillin (39.03%) and tetracycline (34.2%) in this study and previous studies conducted in Iran, resistance to these antibiotics has varied across different regions in recent years. And it is generally increasing. However, the level of resistance to these antibiotics is lower compared to metronidazole and clarithromycin antibiotics. Therefore, the use of these antibiotics in the first-line treatment of *H. pylori* is suggested to achieve better results in the treatment and eradication of this bacterium in Iran.

## Conclusion

 The prevalence of *H. pylori* infection strongly depends on the geographical area in which the patients reside. Epidemiological studies enable physicians to narrow down their antibiotic choices and make more cautious antibiotic prescribing decisions based on the overall picture of *H. pylori* in their areas. Therefore, it is recommended to conduct such studies to obtain a comprehensive antibiotic profile and use the appropriate treatment line.
